# Lord Berkeley's Letter to Dr. Jenner, on the Cow-Pox

**Published:** 1801-08

**Authors:** 

**Affiliations:** Cranford Lodgs


### Lord BERKELEY'S *Letter to* DR. JENNER, *on the Cow-Pox.*

MY DEAR SIR,

YOU will doubtless be much pleased to hear of a circumstance that has happened in my family, to confirm, in the most substantial manner, the power the Cow-pox possesses of preventing the Small-pox. One of my house-maids was so unfortunate, some weeks ago, as to catch the Small-pox, and after struggling with it for upwards of three weeks in my house here, she at last became its victim. My youngest son, you know, was inoculated by you about a year ago with Cow-pox, and about the same time, a maid servant, by Mr. Shrapnell, surgeon to my regiment; and another, upwards of four years back, by some one in the country. As soon as matter could be procured from the ſervant girl with the Small-pox, my ſon, and one of the girls who had previouſly had the Cow-pox, were inoculated. The inoculated part looked red and angry, as if it would feſter, for a few days, but then died away, without producing any effect; the other girl was repeatedly expoſed to the infection, without inoculation, *during the whole progreſs of the diſorder*, but has not felt any thing from it. I did not inoculate this laſt mentioned girl, from a certainty in my own mind of the impoſſibility of giving the Small-pox when the Cow-pox had been properly given, having myſelf been witneſs for theſe fifteen years paſt to your perſevering labours, which at laſt have attained ſuch perfection, that in a few years the Small-pox muſt be eradicated, and thouſands of your fellow-creatures, annually, will owe their lives to your diſcovery.

I may add, that the child and the other ſervant who were inoculated, were alſo expoſed to infection.

Theſe facts appear to me ſo very ſtriking, that I could wiſh you to make them public in any way you may think proper.

I am,

My dear Sir,

Your moſt obedient ſervant,

BERKELEY.

*Cranford Lodge,*

*July* 4, 1801.

P. S. Allow me to mention, that there is an old ſervant in my family, on the verge of ſeventy, who had the Cow-pox from milking cows, when a boy. From that time he has never been in the leaſt cautious in guarding himſelf from the Small-pox, but has expoſed himſelf again and again, without being ſenſible of its effects. I mention this circumſtance, becauſe, in converſation on the ſubject, I ſometimes find that the Cow-pox is ſuppoſed to be a *temporary ſecurity only* againſt the Small-pox.

